# Editorial: Interpretable and explainable machine learning models in oncology

**DOI:** 10.3389/fonc.2023.1184428

**Published:** 2023-03-22

**Authors:** William Thomas Hrinivich, Tonghe Wang, Chunhao Wang

**Affiliations:** ^1^ Department of Radiation Oncology and Molecular Radiation Sciences, Johns Hopkins University, Baltimore, MD, United States; ^2^ Department of Medical Physics, Memorial Sloan Kettering Cancer Center, New York, NY, United States; ^3^ Department of Radiation Oncology, Duke University, Durham, NC, United States

**Keywords:** explainable machine learning, oncology, explainable deep learning, radiotherapy, MRI, outcome prediction, ultrasound

The application of machine learning (ML) in cancer diagnosis and treatment is rapidly expanding, capitalizing on the availability of highly-detailed patient-specific data, advancements in hardware performance, and algorithmic breakthroughs. ML approaches have been applied to all facets of oncology (1) including medical image reconstruction (2) and classification (3), interpretation of histopathology (4, 5), genomic analysis (6), chemotherapy and radiotherapy outcome prediction (7; 8) and surgical guidance (9). As described by Lu et al. in this collection, model “explainability” may refer to the ability to describe elements of the model and “interpretability” may refer to the ability to understand reasoning behind the model’s prediction. ML model explainability and interpretability (MEI) are of growing interest (10) as models grow in complexity through highly non-linear approaches such as deep learning (DL), which may offer unparalleled performance but provide little or no inherent MEI (11).

Limited MEI associated with sophisticated ML approaches creates concerns for investigators and clinicians (12). In situations when ML models are used to guide critical decision making for a patient’s cancer care, poor MEI may introduce safety concerns preventing clinical adoption and degrading overall trust in model efficacy. Limited MEI may prevent human users from making informed decisions regarding the relevance of a model’s output to a specific scenario or from supplementing a prediction with their existing knowledge. These issues are evidenced by instances where large models provided predictions based on extraneous features unrelated to patient clinicopathological or physiological features due to biases in training data (13). Finally, limited MEI poses challenges for investigators pursuing improved performance by hindering interpretation of factors impacting model accuracy and generalizability.

Accordingly, many approaches have been proposed to improve MEI in oncology and other domains through a variety of approaches that may be considered model-specific or model-agnostic, and *ad-hoc* or *post-hoc*. Lu et al. (11, 14, 15), Model-specific approaches are only applicable for specific models, such as certain saliency map approaches for convolutional neural networks (16). Model-agnostic approaches may be applied more generally, often numerically investigating the relationships between model inputs and outputs (17). *Ad-hoc* methods include approaches intended to make the model intrinsically explainable and may include hand-crafted feature selection or the incorporation of guiding heuristics based on existing physics on oncologic principles. Alternatively, *post-hoc* methods are those that may be applied following model design and training. Improved MEI through these and other methods hold promise to promote the safety and quality of ML models in oncology as approaches grow in complexity, thereby improving synergy with clinicians and leading to real-world improvements in patient care. This collection includes five articles covering the following themes:

## MR image reconstruction using patient-specific prior

MR imaging plays a critical role in many facets of oncology including diagnosis, treatment, and response assessment. When applied to advanced radiotherapy guidance through MRI-guided linear accelerators, acquisition time is of critical importance to reduce treatment time and mitigate effects of patient motion. Moreover, a high level of trust and comprehensive understanding of image features are imperative to facilitate critical therapeutic decisions. Grandinetti et al. propose a DL-based approach to enable high-quality reconstruction of under-sampled MR images enabling significant reduction in acquisition time. The authors propose incorporating patient-specific regularization using fully sampled pre-treatment diagnostic MRI of the same patient, providing the end user with an interpretable model overcoming limitations in population-average data. Using this approach, the authors demonstrate the ability to achieve high quality image reconstruction with significantly under-sampled data in both phantoms and patient cases.

## Ultrasound-based prostate segmentation using an interpretable model expression

Trans-rectal ultrasound (TRUS) is commonly employed for the localization and guidance of needles for prostate biopsy and delivery of therapies including brachytherapy. In the case of ultrasound brachytherapy, localization and delineation of the needles and prostate gland are critical for effective treatment planning and delivery. In this application, time-efficiency is also critical since all steps must be completed while the patient is anesthetized. Peng et al. propose a semi-automatic prostate segmentation approach using ML and a principle curve based on an interpretable mathematical model expression. In this case, an ML model is combined with human initialization to create seed points, thereby providing flexibility for human input while augmenting results with an ML model providing the user with understanding and control of the algorithm output. The authors demonstrate improved prostate segmentation accuracy on patient images compared to existing state-of-the-art algorithms.

## Cancer detection using non-invasive behavioral data

Several ML approaches have been proposed to aid in the early detection of cancer based on non-invasive data such as self-reported lifestyle characteristics, weight, or heart rate. However, ML models derived from large patient cohorts with potentially noisy or extraneous features may preclude interpretability by end users. Jiang et al. describe an ML model trained to predict gastric cancer diagnosis based on non-invasive lifestyle characteristics such as age, smoking history, and family cancer history. To ensure interpretability, the authors choose decision tree classifiers enabling *post hoc* analysis of input feature importance, demonstrating that XGBoost provided the highest prediction performance in the test cohort. Feature importance was reported, demonstrating that the top 5 features matched with those reported as predicted in previous prospective studies.

## Outcome prediction using a human-in-the-loop-based Bayesian network approach

Outcome prediction for hepatocellular carcinoma (HCC) patients following stereotactic body radiotherapy (SBRT) remains challenging due to limitations in curating training databases and overcoming imbalances in outcomes, which can lead to biased prediction results. Luo et al. propose a “human-in-the-loop” (HITL) based Bayesian network approach to mitigate these challenges by including input from human experts during the selection of clinical features derived from HCC patients to create the prediction model of post-SBRT albumin-bilirubin grades, rather than relying on purely algorithmic feature selection which may suffer from biases in the training data. The HITL was found to not only improve interpretability of the final models, but also outperformed a purely data-driven approach in an independent test cohort from an outside institution.

## Review of interpretable ML predictions for decision making in oncology

Following the application-specific investigations with considerations for MEI in the preceding four articles, Lu et al. conduct a thorough discussion and review of the challenges and importance of MEI in oncology, and an overview of algorithms and strategies applied to interpretation of complex non-linear ML models. The authors demonstrate the application of these algorithms to identify cancerous breast masses using a publicly available dataset of cell nuclei characteristics, highlighting strengths and limitations of each approach. The authors conclude that MEI is an important consideration in oncology, and that many strategies exist to probe even highly complex and non-linear ML models, potentially leading to improvements in both performance and clinical adoption.

## Author contributions

CW and TW are the coeditors for this Research Topic. WH is the review editor for this Research Topic. All authors contributed to the article and approved the submitted version.
